# Integration of Prevention of Mother-to-Child Transmission of HIV (PMTCT) Postpartum Services with Other HIV Care and Treatment Services within the Maternal and Child Health Setting in Zimbabwe, 2012

**DOI:** 10.1371/journal.pone.0098236

**Published:** 2014-06-10

**Authors:** Katherine Wiegert, Thu-Ha Dinh, Angela Mushavi, Owen Mugurungi, Peter H. Kilmarx

**Affiliations:** 1 US Centers for Disease Control and Prevention -Hubert Global Health Fellow, Atlanta, Georgia, United States of America; 2 Duke University Medical Center, Durham, North Carolina, United States of America; 3 US Centers for Disease Control and Prevention, Center for Global Health, Division of Global HIV/AIDS, Atlanta, Georgia, United States of America; 4 AIDS & TB Unit, Ministry of Health & Child Care of Zimbabwe, Harare, Zimbabwe; 5 US Centers for Disease Control and Prevention, Center for Global Health, Division of Global HIV/AIDS, Harare, Zimbabwe; University of Washington, United States of America

## Abstract

**Background:**

We assessed the integration of PMTCT services during the postpartum period including early infant diagnosis of HIV (EID) and adult and pediatric antiretroviral therapy (ART) in maternal and child health (MCH) facilities in Zimbabwe

**Methods and Findings:**

From August to December 2012 we conducted a cross-sectional survey of a nationally representative sample of 151 MCH facilities. A questionnaire was used to survey each site about staff training, dried blood spot sample (DBS) collection, turnaround time (TAT) for test results, PMTCT services, and HIV care and treatment linkages for HIV-infected mothers and children and HIV-exposed infants. Descriptive analyses were used.

Of the facilities surveyed, all facilities were trained on DBS collection and 92% responded. Approximately, 99% of responding facilities reported providing DBS collection and a basic HIV-exposed infant service package including EID, extended nevirapine prophylaxis, and use of cotrimoxazole. DBS collection was integrated with immunisations at 83% of facilities, CD4 testing with point-of-care machines was available at 37% of facilities, and ART for both mothers and children was provided at 27% of facilities. More than 80% of facilities reported that DBS test results take >4 weeks to return; TAT did not have a direct association with any specific type of transport, distance to the lab, or intermediate stops for data to travel.

**Conclusions:**

Zimbabwe has successfully scaled up and integrated the national EID and PMTCT programs into the existing MCH setting. The long TAT of infant DBS test results and the lack of integrated ART programs in the MCH setting could reduce effectiveness of the national PMTCT and ART programs. Addressing these important gaps will support successful implementation of the 2014 Zimbabwe's PMTCT guidelines under which all HIV-infected pregnant and breastfeeding women will be offered life-long ART and decentralized ART care.

## Introduction

HIV infections continue to be a source of significant mortality in the pediatric population. As of 2012, approximately 3.3 million children younger than 15 years were living with HIV worldwide, and there were 260,000 new infections in children [1]. Mortality is high among HIV-infected infants in their first year of life; approximately 30% of HIV-positive children do not survive to their first birthday, and half die before the age of two years [2]. Mother-to-child transmission of HIV (MTCT) is by far the most common source of pediatric HIV infection, responsible for greater than 90% of new HIV infections among infants [3]. In an effort to reduce mortality of children less than five years of age, the World Health Organization (WHO), United Nations and AIDS (UNAIDS) and United Nations Children's Fund (UNICEF) with supports from other international health partners, have called for eliminating HIV infections in children and keeping their mothers alive. Prevention of mother-to-child-transmission (PMTCT) interventions can reduce MTCT to less than 5% [4].

In Zimbabwe, a country that saw a decline in HIV prevalence from 22.8% to 14.7% between 2002 and 2012 [5], HIV and acquired immunodeficiency syndrome (AIDS) remain the single leading cause of childhood mortality [6]. Thus, the National PMTCT Programme added an early infant diagnosis (EID) program using HIV deoxyribonucleic acid (DNA) polymerase chain reaction (PCR) testing to the package of comprehensive PMTCT services in 2007 [7]. The Ministry of Health and Child Care (MOHCC) in Zimbabwe, in an effort to reduce MTCT of HIV from an estimated 30% in 2009 to <5% by 2015 [8], adopted the 2010 WHO/PMTCT Option A recommendations. For pregnant women with CD4 ≤350, antiretroviral therapy (ART) is provided for life, and for pregnant women with CD4 >350 zidovudine is provided during pregnancy and single-dose nevirapine (sdNVP) at delivery, followed by daily nevirapine (NVP) syrup for infants throughout the duration of breastfeeding. The WHO and UNAIDS has identified that decentralizing and integrating HIV care and treatment services with other areas of health care, such as maternal and child health services as a key strategy for maximizing access to these services [9]. In Lusaka, Zambia, integration of ART into existing public sector maternal and child health clinics doubled the proportion of women initiating ART [10]. It has been shown that integration of HIV services with other clinic-based services such as antenatal care, nutritional services, and family planning positively affects uptake of ART [10], [11], and reduces time to treatment initiation [12]. Rollins et al. showed that EID integration can be effective when combined with immunization clinics [13]. A recent Cochrane review concluded that integration is feasible and is promising in improving health and behavioural outcomes toward HIV treatment and care; however, gaps remain in research to determine effectiveness of integration [14].

We conducted a rapid assessment to review the integration between PMTCT services providing to HIV-exposed infants, including EID programs, and HIV care and treatment for HIV-infected mothers and children during postpartum period in the primary MCH facilities in Zimbabwe

## Methods

### Study design

Between August and December 2012, we conducted a cross-sectional survey of a nationally representative sample of public MCH facilities providing routine MCH care services. Using a structured questionnaire, data were collected from 151 facilities through phone interviews; self-administrative questionnaires sent through mail, email or delivered in person.

### Sampling frame

Facilities surveyed included primary health care facilities, district hospitals, mission/rural hospitals, and provincial hospitals. Primary health care facilities provide basic outpatient services at the entry level of care. District/mission hospitals are comprised of government designated hospitals in districts without a government hospital, where in addition to primary care, emergency services are provided. Provincial hospitals constitute the highest referral level in the province including specialists in different medical disciplines [6]. Of 1,542 public MCH facilities, we selected 1,007 facilities providing ≥100 of the 1^st^ diphtheria-pertussis-tetanus (DPT1) vaccinations to infants in 2011 in Zimbabwe. Facilities providing <100 DPT1 vaccinations (representing 8.6% (n = 535) of the total infants receiving the 1^st^ DPT in 2011) were excluded due to resource and logistical considerations. Using quartile distribution of the number of DPT1 vaccinations providing by the eligible facilities in 2011, the 1007 eligible facilities were originally grouped into four groups, facilities providing 100–140 the 1^st^ DPT, 141–235, 236–329, and ≥330 the 1^st^ DPT vaccinations. Since services providing at facilities in the 2^nd^ and 3^rd^ quartile were similar, we combined these two groups into one group. To ensure the representation of participating facilities from each stratum including the combined group of the 2^nd^ and the 3^rd^ quartile, we randomly selected 15% of eligible facilities from each stratum; thus, a total of 151 facilities from all 10 provinces in Zimbabwe were included in our final facility sample.

### Data collection tools

The standardized paper questionnaire (English version), which was piloted in five different facilities, was mailed to the head nurse at each selected facility with a request to return it within two weeks. For those sites that did not complete the questionnaire within 2 weeks, a data collector interviewed the clinic nurse over the phone with the same questionnaire. If a site was unreachable by mail, then by phone after four attempts, the interview was conducted at the site. On average the questionnaire took 15–20 minutes to complete.

The questionnaire instrument surveyed each selected facility about human resources, staff training needs for conducting HIV testing in children <2 years of age, infant DBS sample transportation from facility to the national laboratory, turnaround time of test results to facility, PMTCT services, postnatal care, and HIV care and treatment linkages for HIV-infected mothers and children and HIV-exposed infants in 2012. Additionally, NMRL was asked to provide information whether DBS were ever submitted from the selected facilities.

### Data analysis

We defined a clinic providing (1) a basic postnatal PMTCT package if at least the following services were provided: DBS sample collection and nevirapine and cotrimoxazole prophylaxis for HIV-exposed infants; and (2) a comprehensive PMTCT and HIV care and treatment package if the following were provided at the same facility: HIV testing and counselling for adults, CD4 count testing on site, and adult and pediatric HIV care and treatment (ART) in addition to the basic postnatal PMTCT package. We defined integration of EID with routine MCH services as the facility offering DBS collection along with immunizations or postnatal visit care.

Since NMRL was the only laboratory where DBS samples are processed for EID and is located in Harare, we estimated distance the DBS samples and results would need to travel to get to NMRL as a potential factor associated with EID test result's turnaround time (TAT). We grouped those facilities located in provinces that border Harare (Mashonaland East, Mashonaland Central, Mashonaland West) as the “close to NMRL” group; and the other facilities were in the “far from NMRL” group.

Turnaround time (TAT), an important determining factor for efficiency of the national EID program, was defined as the time from the day the DBS sample was collected to the day that HIV DNA PCR results are returned to the facility. Methods of transport were also analysed for potential associated factors with TAT for DBS results. We grouped MOHCC, the City Health Department, and ambulance into a group called “internal transport” and other public transport and courier transport into “public methods.”

Descriptive and logistic regression analysis methods were used. Data were analysed using SAS Enterprise Guide software version 4.3 (SAS Institute Inc. 2011. Base SAS 9.3 Procedures Guide. Cary, NC: SAS Institute Inc.).

The assessment was approved as part of the evaluation of the effectiveness of the national PMTCT program in Zimbabwe (ZWPMTCTE) by the institutional review board of the Zimbabwe Medical Research Council and the Office of Associate Director of Science at the United States Centers for Disease Control and Prevention (CDC). Since the assessment did not obtain sensitive information or personal opinions, clinic nurses provided verbal consent to participate in the assessment. Of those who refused to participate in the assessment, we documented in the database as “Not complete” and their clinics were still eligible to participate in the evaluation of ZWPMTCTE.

## Results

**Figure 1:**
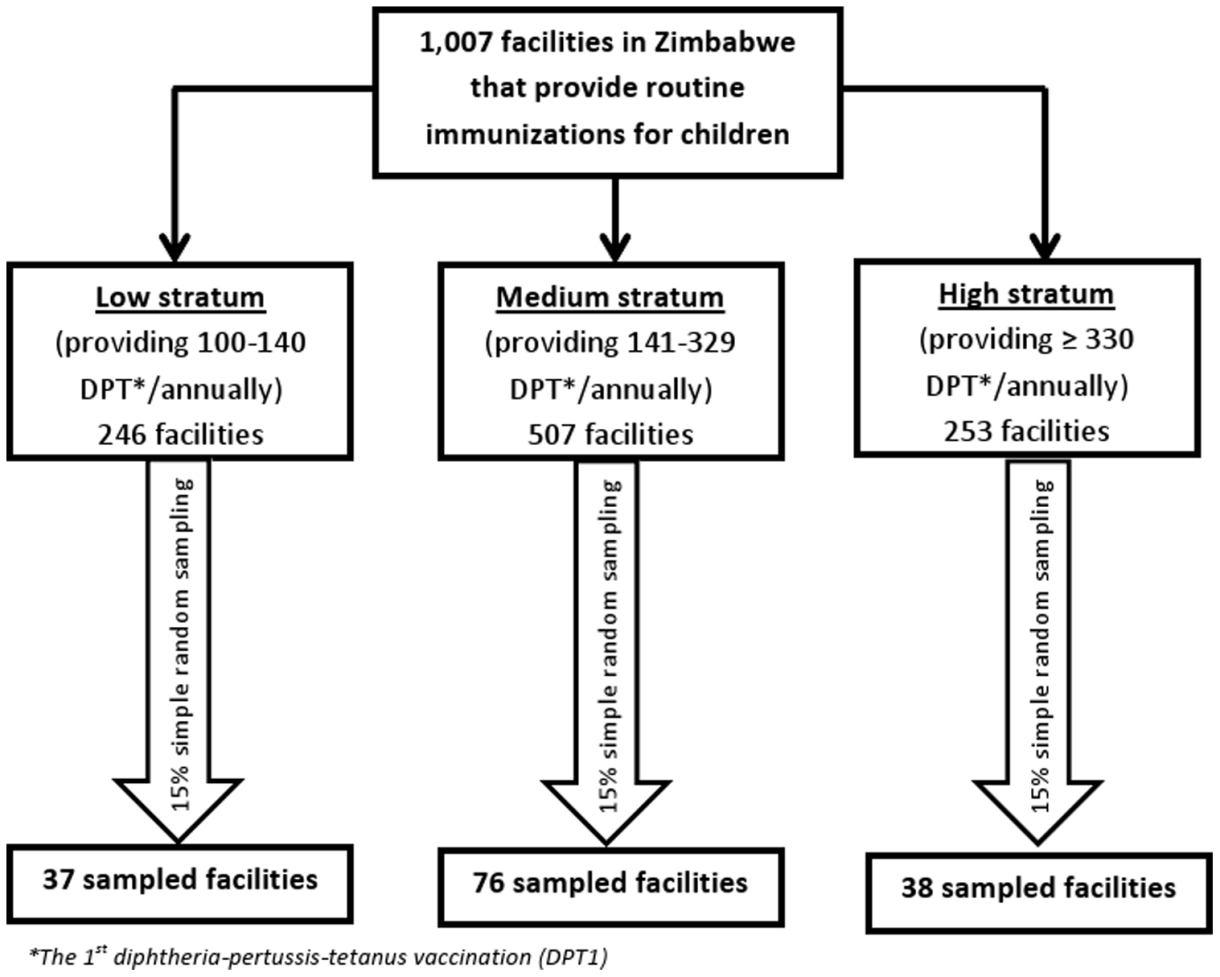
Sampling of Facilities and Response Rate by Stratum. DPT: The 1^st^ diphtheria-pertussis-tetanus vaccination.

Out of the 151 selected facilities, 139 (92%) completed the survey with 55 by mail, 79 over the phone, and five in person; 12 (8%) facilities did not complete the survey (Figure 1). Most of the non-response facilities were in medium- and high-patient volume facilities (Figure 1). Table 1 shows that 85% (n = 118) of the responding selected facilities were primary health care facilities, 5% were district hospitals, 9% were mission or rural hospitals and 1% were provincial hospitals.

**Table 1:** Service provision model in integrating EID, PMTCT, HIV/AIDS care and treatment into MCH setting during post-delivery period in Zimbabwe, 2012 (N = 139).

Characteristic	N(%)*	Stratum level of facility volume; N (%)of facilities that provide the serviceδ		
		Low¤	Medium^t^	High¥
**Facility type**				
Primary health care	118 of 139 (85%)			
District Hospital	7 of 139 (5%)			
Mission/Rural Hospital	13 of 139 (9%)			
Provincial hospital	1 of 139 (1%)			
**Staff**				
Training on EID	139 of 139 (100%)			
Training and provide EID	138 of 139 (99%)			
**Service provision** δ				
DBS collection	138 of 139 (99%)	36 of 36(100%)	67 of 68 (98%)	35 of 35(100%)
HIV testing and counselling for mothers	123 of 137 (90%)	33 of 35 (94%)	57 of 67 (85%)	33 of 35 (94%)
POC CD4 testing machine on site	49 of 136 (36%)	7 of 33 (21%)	26 of 68 (38%)	16 of 35 (46%)
Adult HIV Treatment	88 of 135 (65%)	18 of 32 (56%)	38 of 68 (56%)	22 of 35 (63%)
Pediatric HIV Treatment	78 of 135 (58%)	22 of 33 (67%)	39 of 67 (58%)	27 of 35 (77%)
**Infant-DBS collection for HIV testing (EID)**				
Provision at the same time with infant's immunizations (integrated)	114 of 138 (83%)			
Package of comprehensive PMTCT and maternal and child HIV care and treatment services(1)	37 of 138 (27%)			

(1) including HIV testing and counselling, DBS collection, nevirapine and cotrimoxazole prophylaxis, CD4 point of care machine on site, and Adult and pediatric HIV treatment.

δ *Varying total N (denominator) due to missing data.*

*percentages add to greater than 100% because facilities provide more than one type of service.

¤ *Facilities with 100–140 yearly DPT visits (low stratum).*

t *Facilities with 141–329 yearly DPT visits (medium stratum).*

¥ *Facilities with 330+ yearly DPT visits (high stratum).*

### PMTCT, EID, and antiretroviral therapy (ART) services for adults and children

Of 139 responding facilities, 100% reported having at least one staff member trained in collecting DBS samples for HIV DNA testing (Table 1), and 98% (n = 136) reported providing postnatal care services. Although DBS collection scheduled at 6 weeks postpartum was reportedly provided in 99% (n = 138) of the facilities, the NMRL report indicated that DBS was submitted from all selected facilities in 2012. The DBS collection service was reported offering in conjunction with the 1^st^ DPT immunisations at 83% (n = 114), and with postnatal visits at 80% (n = 111) of the responding facilities.

HIV testing and counselling for adults including men and women also were reported to be provided at most (90%) of responding facilities. On-site CD4 testing (using a point-of-care machine) was offered to HIV-infected patients in 21% of low-volume facilities, 38% of medium-, and 46% of high-volume facilities. There were no detectable differences between the three strata in any other category of service provision. Pediatric and adult HIV treatment were also not universal; however, more facilities offered adult HIV treatment (65%) than pediatric HIV treatment (58%) (p = 0.012).

Although providing a basic postnatal PMTCT service package including EID, extended nevirapine prophylaxis during the breastfeeding period, and cotrimoxazole to HIV-exposed infants, was reported in 99% of the responding facilities, the comprehensive package for postnatal PMTCT including HIV care and treatment services (ART) for adults and children was provided at only 27% (n = 37) of the facilities (Table 1). CD4 testing or HIV treatment for children or adults necessitated referral in 73% (n = 121) of facilities. However, at the referral facilities HIV care and treatment services were usually available to both adults and children at the same facility except in one case. That single case was in a city where the referral clinics were not far from each other.

A percentage of facilities reported needing to refer for the following services: 10% (14) for HIV testing and counselling, 63% (87) for CD4 testing, 42% (57) for pediatric care and treatment, 35% (47) for adult HIV care and treatment. Estimated distance to referral site from primary facility site is described in table 2. The median distance that referred patients may have to travel to reach HIV testing and counselling is 33 km (range, 1.5–60 km), to reach pediatric and adult care and treatment services was 17 km (range, 1.5–140 km), and to reach CD4 testing facilities was 24.5 km (range, 0.5–140 km).

**Table 2:** Referral services needed and distance from the primary facilities (N = 121).

Service referred (N = 121)	Number of facilities	Median distance to referral, range [standard deviation]	N (%) with Distance between the primary facility to Referral Site (km)		
			0–10	10–20	>20
HIV testing and counselling for adult	14	33 km (1.5–60 km) [10.8]	3 (21.4%)	3 (21.4%)	8 (57.2%)
CD4 testing	66	24.5 km (0.5–140 km) [25.8]	19 (28.8%)	11 (16.7%)	36 (54.5%)
Pediatric HIV Treatment	43	17 km (1.5–140 km) [22.4]	18 (41.9%)	9 (20.9%)	16 (37.2%)
Adult HIV Treatment	33	17 km (1.5–140 km) [22.0]	13 (39.4%)	5 (15.2%)	15 (45.5%)

### EID (DBS) test result turnaround time

Only 19% of facilities reported a TAT of less than 4 weeks, although 71% sent DBS samples weekly to the NMRL for DNA PCR testing. The collected DBS samples were sent to the NMRL either directly (15%), through intermediaries at the provincial level (13%), or through intermediaries at the district level and then provincial levels (72%). The NMRL generally sent the test results to facilities using the same route in which the DBS were sent to them.

Bivariate analysis (Table 3) suggests that methods of transporting DBS samples to NMRL have no association with TAT (p>0.05). We also found that how DBS samples get sent to the laboratory (directly vs. through intermediary), frequency of sending DBS samples, proximity of province to NMRL, patient volume, and number of transport methods utilized were not associated with TAT in bivariate analysis. Only when stratified by closeness to the laboratory, utilizing MOHCC (p<0.05) and environmental health technicians (EHT) (p<0.0005) were associated with shorter TAT in the facilities in provinces closer to the NMRL (not included Table 3).

**Table 3:** Bivariable analysis: Factors associated to turnaround time for DBS HIV DNA PCR testing.

Characteristic	N	% with Turnaround time <4 weeks	P value*
**Transport DBS/results with internal transport (MOHCC, CityHD, and ambulance)**			0.341
Yes	112	20	
No	23	13	
**Transport DBS/results with public methods (public transport, courier)**			0.526
Yes	79	19	
No	56	18	
**Transport DBS/results with Environmental Health Technicians**			0.097
Yes	23	30	
No	112	16	
**Where the DBS get sent**			
No intermediate stops–Send directly to the National Microbiology Reference Laboratory (NMRL)	21	24	0.341
One intermediate stop—sending to provincial hospital	17	18	0.650
Two intermediate stops—sending to district hospital	98	18	0.634
**Frequency of sending DBS**			0.420
At least weekly	96	20	
Less frequent than weekly	37	16	
**Facility in province close to the NMRL**			0.775
Yes	55	16	
No	80	20	
**Facility volume stratum**			0.709
Low (100–140 yearly DPT visits)	35	23	
Medium (141–329 yearly DPT visits)	67	18	
High (330+ yearly DPT visits)	33	15	
**Number of transport methods utilized**			0.555
1	40	18	
2	63	16	
3–5	32	25	

*Using Fisher's exact test one sided.

None of the factors proved to be statistically significant associated with TAT in a logistic regression model that included distance from province to NMRL, frequency of sending DBS, where DBS get sent, number of transport methods utilized, using MOHCC transport for DBS or results, and using EHT for transport of DBS or results.

## Discussion

The results of this first assessment using a national representative sample of public facilities in Zimbabwe. The assessment provides an overall picture of success of implementing a national-scale EID programme, integrating PMTCT services, and delivering HIV care and treatment for mothers and infants during post-delivery within the public MCH setting in Zimbabwe as of December 2012. Furthermore, our findings provide identified gaps that need to be addressed in order to successfully scale-up the national ART as well as roll-out the national PMTCT option B+ which started in January 2014.

It is encouraging that 100% of sampled facilities had staff trained in collecting DBS from infants for HIV testing and that DBS sample collection was offered at 99% of sampled facilities in 2012, which surpasses the MOHCC goal of 95% by 2015 [6]. In addition, our findings show that basic PMTCT services for HIV-exposed infants were provided at almost all facilities (99%). Furthermore, EID is integrated with other routine services at the same location which were offered to infants such as immunizations and postnatal visits in the majority of facilities, 82% and 80%, respectively. This integration will potentially provide opportunities to reduce loss to follow-up [15], allowing early initiation of treatment for HIV-infected infants as well as further preventing MTCT during breastfeeding, which may reduce infant mortality from HIV [16]. However, only 58% of facilities offered ART for HIV-infected children and adults including mothers, with referral facilities located, on average, 17 km (range, 1.4 to 140 km) away from the primary facility. For women with no transport options this may be an insurmountable challenge, contributing to loss to follow-up secondary to poor linkages in the system. Although Zimbabwe has adopted the 2013 WHO PMTCT recommendations in which all HIV-positive pregnant women will be on ART for life (Option B+) since January 2014, knowing a mother's CD4 count may not be urgently but is needed to monitor treatment. With less than 41% and 21% of clinics at medium/high and low patient volume facilities, respectively, providing CD4 testing with a point-of-care machine on site, potential for monitoring ART treatment may be negatively affected. The median distance travelled for CD4 testing if not provided at the site is 24.5 km, and this distance to receive testing could impact determining of switching ART regimens in a timely fashion.

With only 65% of facilities provide ART to all adults including pregnant and breastfeeding women by 2012, Zimbabwe will face challenges in rolling out the PMTCT option B+ and reaching the national goal of increasing infant ART provision coverage to 90% of all facilities by 2015. The lack of ART integration with services in the MCH setting for both mother and infant may increase the risk of infant/maternal mortality due to loss to follow-up for those who need treatment [15].

Turnaround time of the HIV DNA test results using infant-DBS as part of the EID services was found to be longer than the recommended 4 weeks at 81% of facilities, and in many cases was >2 months. Since we could not find any factors that were statistically significant associated with TAT, we suspect that one main delay of the TAT could be lack of capacity at NMRL, the only public laboratory that could perform HIV DNA PCR in Zimbabwe at the time of the survey. Ongoing efforts to expand the NMRL to one or two more regional/satellite laboratories may help improve the TAT. The delay in receiving results is one contributor to decreased rates of early initiation of treatment for HIV-infected infants to higher infant mortality [17]. Promptness of infant HIV testing and linkage to prophylaxis and treatment initiation are essential; it was shown by Violari et al. (2008) that EID and early initiation of ART therapy at a median age of seven weeks significantly reduces infant mortality and progression of HIV compared to delayed initiation [16]. EID and HIV service uptake may be improved when the complete package of essential services for quality maternal care, including HIV testing and counselling, treatment, and care services, are easy to access and are integrated into existing clinic functions [14], [18], [19]. The improved integration of EID with combined HIV service packages as well as routine clinic services could potentially increase ART uptake and decrease maternal mortality from HIV, in turn reducing the likelihood that infants become AIDS orphans.

This rapid assessment has some limitations. Our findings may not be representative for small facilities providing <100 DPT1 immunizations annually since we excluded them from our sampling frame. Although our response rate was relatively high (93%) and sampling frame was capturing a national representative sample of public MCH facilities in Zimbabwe, our sample size of 139 facilities may be moderately small to measure some specific outcomes of the integrations. Our findings were also mostly based on self-report which could have led to recall bias and social desirability bias in the interview methods. The different methods used to administer the survey, including self-administration and interviewer-administration might have influenced reporting depending on the method utilized. Although our findings provide critical descriptive baseline information on integrations between PMTCT and HIV care and treatment programs just before the PMTCT option B+ was rolling out, future studies should directly measure outcomes of these integrations such as coverage of the 6-week EID, retention rates in care and treatment, as well as maternal and child survival.

Zimbabwe has successfully scaled and integrated the national EID and PMTCT programs into the existing MCH primary health care setting among facilities that provide ≥100 DPT1 vaccinations to infants. However, the long TAT of EID (DBS) test results, and the lack of decentralized and integrated ART programs in the MCH primary health care setting could reduce the effectiveness of the national PMTCT as well as ART programs. Addressing these important gaps will support continual improvement of the EID system, and ensure the success of adopting the 2013 WHO PMTCT recommendations started in January 2014 in which HIV-infected pregnant and breastfeeding women will receive ART in Zimbabwe. Addressing the important gaps identified in this assessment will be necessary to reach the elimination MTCT goals and keeping mothers and babies alive in Zimbabwe.
